# Exploring New Scaffolds for the Dual Inhibition of HIV-1 RT Polymerase and Ribonuclease Associated Functions

**DOI:** 10.3390/molecules26133821

**Published:** 2021-06-23

**Authors:** Rita Meleddu, Angela Corona, Simona Distinto, Filippo Cottiglia, Serenella Deplano, Lisa Sequeira, Daniela Secci, Alessia Onali, Erica Sanna, Francesca Esposito, Italo Cirone, Francesco Ortuso, Stefano Alcaro, Enzo Tramontano, Péter Mátyus, Elias Maccioni

**Affiliations:** 1 Department of Life and Environmental Sciences, University of Cagliari, Cittadella Universitaria di Monserrato, Monserrato, 09042 Cagliari, Italy; rita.meleddu@unica.it (R.M.); angela.corona@unica.it (A.C.); s.distinto@unica.it (S.D.); cottiglf@unica.it (F.C.); serenella.deplano@unica.it (S.D.); lisa.sequeira@unica.it (L.S.); daniela.secci92@gmail.com (D.S.); aleonali95@gmail.com (A.O.); ericasannab@gmail.com (E.S.); francescaesposito@unica.it (F.E.); italo.cirone93@gmail.com (I.C.); tramon@unica.it (E.T.); 2 Dipartimento di Scienze della Salute, Università Magna Graecia di Catanzaro, Campus ‘S. Venuta’, Viale Europa, 88100 Catanzaro, Italy; ortuso@unicz.it (F.O.); alcaro@unicz.it (S.A.); 3 Institute of Digital Health Sciences, Faculty of Health and Public Services, Semmelweis University, Ferenc tér 15, 1094 Budapest, Hungary; peter.maty@gmail.com

**Keywords:** HIV-RT, ribonuclease H, dual inhibitors, docking, putative binding

## Abstract

Current therapeutic protocols for the treatment of HIV infection consist of the combination of diverse anti-retroviral drugs in order to reduce the selection of resistant mutants and to allow for the use of lower doses of each single agent to reduce toxicity. However, avoiding drugs interactions and patient compliance are issues not fully accomplished so far. Pursuing on our investigation on potential anti HIV multi-target agents we have designed and synthesized a small library of biphenylhydrazo 4-arylthiazoles derivatives and evaluated to investigate the ability of the new derivatives to simultaneously inhibit both associated functions of HIV reverse transcriptase. All compounds were active towards the two functions, although at different concentrations. The substitution pattern on the biphenyl moiety appears relevant to determine the activity. In particular, compound 2-{3-[(2-{4-[4-(hydroxynitroso)phenyl]-1,3-thiazol-2-yl} hydrazin-1-ylidene) methyl]-4-methoxyphenyl} benzamide bromide (**EMAC2063**) was the most potent towards RNaseH (IC_50_ = 4.5 mM)- and RDDP (IC_50_ = 8.0 mM) HIV RT-associated functions.

## 1. Introduction

**Figure 1:**
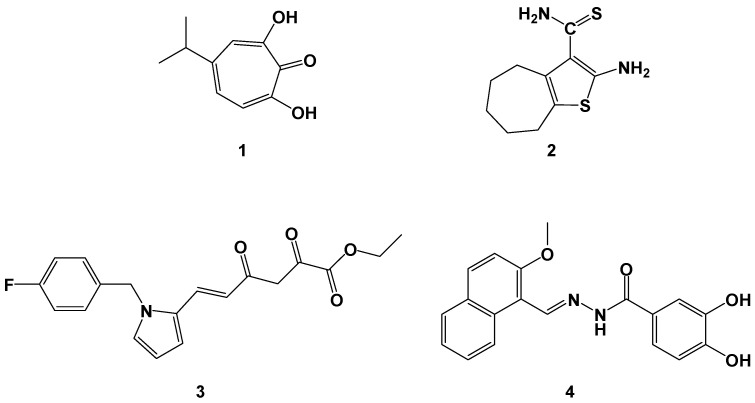
Examples of different RNase H inhibitors: β-thujaplicinol (**1**), vinylogous urea (**2**), β-ketoester (**3**), and hydrazone (**4**).

Since the identification of HIV as the causative agent of the acquired immunodeficiency syndrome (AIDS) [1,2], we have assisted in an impressive drug discovery rush. In less than 30 years this incredible scientific effort has led to the identification of a complex and variegated new armamentarium of antiviral agents, that specifically target HIV [3,4,5,6]. Nowadays, we have several classes of antiviral agents, targeting specific phases of the HIV 1 replication cycle. Hence, since the introduction of azidothymidine (AZT) [7] as the first nucleoside reverse transcriptase inhibitor (NRTI), non-nucleoside reverse transcriptase inhibitors (NNRTI) [8], protease inhibitors (PI) [9], integrase inhibitors (INI), and entry inhibitors (EI) have been approved for clinical treatment of HIV infection [10,11,12]. The current treatment of infected patients always consists of a combination of two or more drugs, according to the presence of viral coinfections and therapy response to the different agents [4,13]. The combination of diverse agents with different targets within the viral replication cycle steps is mandatory, due to the likely selection of resistant mutants [5]. However, it should be considered that, although very efficient in managing the infection, the current therapeutic strategies are not capable of eradicating the virus from the host. For this reason, the current therapeutic protocols are life-long poly-pharmacological therapies that present more than one drawback. In particular, even though simplified therapies (one pill two agents) have been introduced, the adherence to treatment and the management of poly-drug-related toxicities are still issues to deal with [14]. In this respect, the identification of a single agent, able to target two or more functions, related to the HIV replication cycle, could represent an advantageous strategy to reduce both the daily number of pills and the additive toxicity of different agents. Moreover, the use of a single agent with two distinct yet concurring therapeutic effects is undoubtedly a winning strategy to overcome drug-drug interactions [15]. The recent contribution of the scientific community in this direction has been recently reviewed [16]. Interestingly, considering the key role of RT in the HIV replication cycle, conjugated hybrid molecules [17] have been investigated to simultaneously target the NRTI and NNRTI pockets [18,19]. Moreover, it should be considered that HIV RT is a multifunctional enzyme with distinct associated functions, RNA-dependent DNA polymerase (RDDDP), DNA-dependent DNA polymerase (DDDP), and ribonuclease H (RNase H) [20]. However, despite the essential role of the RNase function in the synthesis of the proviral genome [21,22,23], no inhibitors targeting this associated function have been introduced in the clinical use so far. Nevertheless, the attention of the scientific community toward this specific anti-retroviral target has been continuously high [24,25,26,27,28,29,30,31,32]. Indeed, several inhibitors of the RNase H function have been investigated differing from structures and mechanisms of action (Figure 1) [27,28,30,33].

Most of the so far identified RNase H inhibitors, such as β-thujaplicinol (**1**), vinylogous ureas (**2**) and β-ketoesters (**3**), act by chelating the Mg^++^ ions in the ribonuclease catalytic site while a different mechanism of actions has been reported for hydrazone derivatives (**4**). These latter compounds most probably possessed an allosteric mechanism of action and did not exhibit complexing activity towards the Mg^++^ ions. Moreover, some of these hydrazone derivatives were able to inhibit both polymerase and RNase H functions, depending on their substitution pattern [30]. Intrigued by this behaviour, we performed a two steps virtual screening (VS) investigation to identify potential dual inhibitors of polymerase and RNase H functions [34]. Compound **46** (numbered as in the original paper) [34], was identified as the most promising hit possessing dual inhibition ability towards RDDP and RNase H functions at micro-molar concentrations, retaining full potency of inhibition against a multidrug-resistant RT variant [35]. From this starting point, we have synthesized several libraries of derivatives intending to optimise the dual activity on HIV RT-associated functions and to further investigate the mechanism of action of these derivatives [36,37,38,39,40,41,42]. Accordingly with our design strategy, none of these new derivatives was able to chelate Mg^++^ ions, indicating that, in all probability, they do not bind the RNase H catalytic site. Moreover, through a concerted combination of site-directed mutagenesis experiments and computational simulations we identified two putative binding sites for our derivatives, one, namely pocket 1, located in the palm region of RT, near the NNRTIs binding site, and a second, namely pocket 2, located in the RNase H domain, below the catalytic RNase H site [37,38,39]. Starting from these findings we have now designed and synthesised a new library of biphenylhydrazo 4-arylthiazoles derivatives **EMAC2056**–**2071**, structurally related with previously reported compounds to investigate their activity toward both associated functions of HIV RT.

## 2. Results and Discussion

**Scheme 1:**
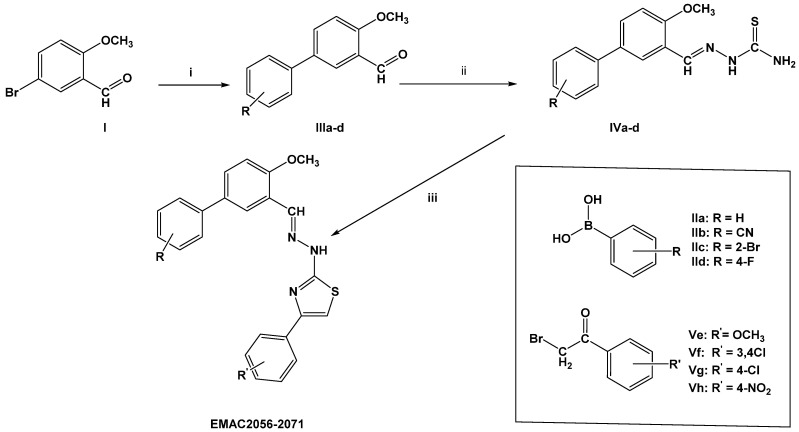
Synthetic pathway to compounds **EMAC2056**–**2071**. Reagents and conditions: (i) DME, Pd(PPh3)4, phenylboronic acid (**IIa**–**d**), aq 2 M Na_2_CO_3_, 110 °C, 24 h. (ii) thiosemicarbazide, *n*-propanol, CH_3_COOH, (iii) α-haloketone (**Ve**–**h**), ethanol, r.t.

Compounds **EMAC2056**–**2071** were synthesized according to the multi-step synthetic pathway depicted in Scheme 1. Namely, the reaction of 5-bromo-2-methoxybenzaldehyde (**I**) with differently substituted phenylboronic acids (**IIa**–**d**) under Suzuki coupling conditions [40] was performed. By this synthetic procedure, intermediate compounds (**IIIa**–**d**) were obtained in good yields ranging from 87 to 98% with the exception of the 2′-bromo-4-methoxy-[1,1’-biphenyl]-3-carbaldehyde (**IIIc**). In this latter reaction the yield was always below 24%. When 2-cyanophenylboronic acid (**IIb**) was used, 3’-formyl-4’-methoxy-[1,1’-biphenyl]-2-carboxamide was formed in the coupling conditions, due to the basic reaction media. The obtained biphenyl aldehydes were then purified and reacted with thiosemicarbazide in *n*-propanol with a catalytic amount of acetic acid. As well as in the precedent synthetic step, yields were satisfactory and generally above 80%. Once more, in the case of the 2-bromo substituted compound (**IVc**) lower yields, around 60%, were observed. To accomplish the cyclisation of the thiosemicarbazones, to form the thiazole ring of compounds **EMAC2056**–**2071**, compounds **IVa**–**d** were reacted with the appropriate α-haloketones (**Ve**–**h**). The reaction might lead to the formation of side products, due to the similar reactivity of the thioureidic nitrogen atoms. However, by performing the cyclization in absolute ethanol at rt, the formation of side products was not observed.

Moreover, reaction yields were generally quantitative and, only in two cases, 2-{4-methoxy-3-[(1*E*)-{2-[4-(4-methoxyphenyl)-1,3-thiazol-2-yl]hydrazin-1-ylidene}methyl] phenyl}benzamide bromide (**EMAC2060**) and 2-{3-[{2-[4-(3,4-dichlorophenyl)-1,3-thiazol-2-yl]hydrazin-1-ylidene}methyl]-4-methoxyphenyl}benzamide bromide (**EMAC2061**), lower than 80%.

All the synthesized compounds structures were confirmed by means of ^1^H-NMR and ^13^C-NMR. **EMAC2056**–**2071** NMR spectra are reported in the materials and methods section.

RT is highly flexible enzyme; therefore, this should be considered for docking experiments [43]. Indeed, NNRTI binding pocket can allow the binding of inhibitors with different shapes and sizes. The shapes of these molecules have inspired medicinal chemists and have been accordingly named as butterfly, horseshoe, and U-shaped conformations [44]. Hence, we carried out QM polarized ligand (QMPL) docking [45] experiments on ensemble HIV-1 RT protein conformations available in the PDB [46]: 1vrt [47], 2zd1 [48], 1ep4 [49], 3qo9 [50], 1rti [47], 1tv6 [51], 3lp2 [52], which consider the different orientations of residues and primer grip β12-β13 hairpin in the NNRTI binding pocket.

QM Polarized Ligand Docking workflow combines docking with ab initio methods for ligand charges calculation within the protein environment. Subsequently, the best poses were subjected to molecular energy minimization to consider the induced-fit protein conformation change that takes place after ligand binding and the effect of implicit water solvation.

**Figure 2:**
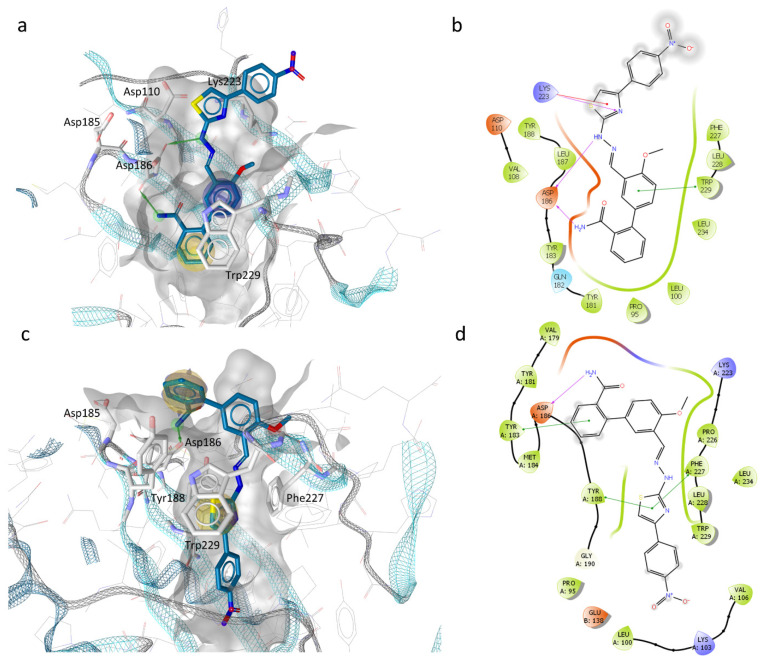
Representation of the putative binding mode of **EMAC2063** obtained by docking experiments. (**a**) 3D depiction of **EMAC2063** and its respective interactions with RT residues considering 3lp2 [52] RT conformation. The yellow sphere indicates hydrophobic interactions with lipophilic residues. While, the Green arrow indicates a hydrogen bond (HB) donor interaction, and the violet sphere represents the aromatic π-π stacking interaction; (**b**) 2D depiction of interactions: the magenta lines indicate HB; the green lines, π-π stacking; the red lines, cation- π interactions; (**c**) 3D depiction of **EMAC2063** and its respective interactions with RT residues considering 2zd1 RT conformation [48]; (**d**) 2D depiction of interactions: the magenta lines, indicate HB; the green lines, π-π stacking.

The best scored binding modes of the most active compound **EMAC2063** are depicted in Figure 2 and show how the compound is predicted to bind in two of the RT conformations considered.

Both binding modes suggest the possible interaction of the compound with polymerase catalytic triad (Asp110, Asp185, and Asp186) and the RNase H allosteric binding site described the first time by Himmel for **DHBNH** [30]. In the first binding mode, the ligand interacts with Lys223, with cation-π interaction, and with Trp229, an important highly conserved residue, with a π-π interaction. Furthermore, the amido group and NH of the hydrazino group interact with Cα and the sidechain of Asp186.

In the second binding mode, the Tyr188 sidechain is rotated in an open conformation which allow a stacking interaction with the thiazole portion. Furthermore, the thiazole is also involved in a T-shape π interaction with Phe227. Finally, the amide group interacts with a hydrogen bond with Asp186.

Overall, the importance of the amide group is clear for this series of compounds.

Hence, from the docking experiments, we can hypothesize that the inhibitory activity could be explained by the short-range inhibition of RDDP activity and a long-range inhibition of RNase H activity, most probably carried out by deviating the nucleic acid trajectory. However, docking experiments do not exclude the possibility that the compound could also bind in the pocket below the RNase H catalytic site, as previously described for other compounds synthesised, but this second hypothesis does not seem to be the preferred one [38].

Compounds **EMAC2056**–**2071** were evaluated for their ability to inhibit RDDP and RNase H RT-associated functions in comparison with efavirenz and b-thujaplicinol, respectively, a known NNRTi and a magnesium binder inhibitor of RNase H. Inhibitory assays were performed on a full length HIV-1 RT group M subtype B, with no pre-incubation with the enzyme as described in the material and methods section. Their activity and RDDP/RNase H ratio is reported in Table 1. All derivatives inhibit both functions with IC_50_ values ranging from 4.5 to 57.0 and 8.0 and 88.0 μM toward RNase H and RDDP, respectively. Within the different substitutions on the biphenyl ring, the 2-carboxamide appeared beneficial for the dual activity. Compounds **EMAC2060**–**2063** were the most globally active with IC_50_ values ranging from 4.5 to 21.5 μM and 8.0 to 27.0 μM towards RNase H and RDDP functions. 2-bromobiphenyl (**EMAC2064**–**2067**) is well tolerated, in particular for the RNase H function inhibition. The unsubstituted biphenyl (**EMAC2056**–**2059**) and the 4-fluorobiphenyl (**EMAC2068**–**2071**) appeared as the substitutions most detrimental to the dual activity. However, if we consider only the RNase H inhibition ability, these latter compounds exhibited a similar behaviour being relatively active only when the unsubstituted biphenyl or the 4-fluorobiphenyl substitutions are accompanied by the introduction of a 4-methoxyphenyl or 4-nitrophenyl moiety in position 4 of the thiazole ring, regarding compounds **EMAC2056**, **EMAC2059**, **EMAC2068**, and **EMAC2071**. Altogether these data highlight that the 2-[2-({4-methoxy-[1,1′-biphenyl]-3-yl}methylidene)hydrazin-1-yl]-4-phenyl-1,3-thiazole scaffold represents a valid starting point for the design of dual inhibitors of HIV RT associated functions.

## 3. Materials and Method

### 3.1. Chemistry

Unless otherwise noted, starting materials, reagents and solvent were obtains from commercial suppliers are reagent grade and were used without purification.

All melting point were determined by the capillary method on a Büchi-540 capillary melting points apparatus (BÜCHI Labortechnik AG, Meierseggstrasse, Switzerland) and are uncorrected.

All samples were measured in DMSO-d6 solvent at 278.1 K temperature on a Bruker AVANCE III spectrometer (Billerica, MA, USA).

In the signal assignments the proton and carbon chemical shifts are referred to the solvent (^1^H: d = 2.49 ppm, ^13^C downfield methyl signal: d = 34.89 ppm, respectively). Chemical shifts are reported in parts per million (ppm, δ units). Coupling constants are reported in units of Hertz (Hz). Splitting patterns are designed as s, singlet; d, doublet; t, triplet; dd, double doublet; m, multiplet; b, broad.

All reactions were carried out with the use of the standard techniques and were monitored by thin-layer chromatography (TLC) on silica gel plates (60F-254, E. Merck, Merck Group, Darmstadt, Germany), and spots were visualised by UV light.

#### 3.1.1. Synthetic Procedures

##### Preparation of 4-Methoxy-[1,1′-Biphenyl]-3-Carbaldehyde (**IIIa**)

5-bromo-2-methoxybenzaldehyde (**I**) was dissolved in DME and Pd(PPh_3_)_4_ was added to the solution at R.T. under argon flow. After stirring the mixture at RT for 10 min, phenylboronic acid and aq 2 M Na_2_CO_3_ solution were added and the reaction mixture was refluxed at 110 °C (oil bath temperature). After 24 hours, when the reaction was completed (monitored by TLC, eluent: *n*-hexane-ethyl acetate 4:1), the reaction mixture was cooled and poured onto ice (100 g). The solution was filtered on Celite and the filtrate was washed with ethyl acetate (100 mL). The aqueous phase was extracted with ethyl acetate (3 × 50 mL).

The combined organic phase layers were washed with water (1 × 40 mL) and dried over Na_2_SO_4_ evaporated under reduced pressure. The red glue residue was purified by flash column chromatography on silica gel (eluent *n*-hexane-ethyl acetate, 5:1). 

The desired compound has a blue fluorescence visible with UV lamp and it is a crystalline white solid: *R*_f_ = 0.82 (exane-ethyl acetate, 1:1); HPLC: 97.336%; m.p. 77 °C–78 °C; yield: 98.8%. ^1^H-NMR (500 MHz, DMSO): δ 3.97 (s, 3H, OCH_3_), 7.34 (d, 1H, Ar-CH, *J* = Hz 8.5), 7.36 (m, 1H, *J* = 8 Hz Ar-CH) 7.46 (t, 2H, *J* = 8 Hz Ar-CH) 7.65 (d, 2H, *J* = 8 Hz Ar-CH), 7.93 (s, 1H, *J* = 2.5 Hz, Ar-CH), 7.98 (d, 1H, *J =* 8 Hz, *J =* 2.5, Ar-CH) 10.4 (s, 1H, CHO). ^13^C-NMR (100 MHz, DMSO): δ 56.2 (1C, OCH_3_), 113.5 (1C, phenyl), 124.3, (1C, phenyl), 125.6 (1C, phenyl), 126.3 (2C, phenyl), 127.4 (1C, phenyl), 129.0 (2C, phenyl), 132.6 (1C, phenyl), 134.5 (1C, phenyl), 138.7 (1C, phenyl), 161.0 (1C, phenyl), 189.1 (1C, aldehyde).

According to this procedure, the following compounds were prepared:

*3′-formyl-4′-methoxy-[1,1′-biphenyl]-2-carboxamide* (**IIIb**). *R*_f_ = 0.29 (exane-ethyl acetate, 1:7); HPLC: 60.27% (36.7% O=PPh_3_) m.p. 140 °C; yield: 87.2%. ^1^H-NMR (500 MHz, DMSO): δ 3.96 (s, 1H, OCH_3_), 7.34 (bs, 1H, NH_2_), 7.29 (d, 1H, *J* = 8.5 Hz, Ar-CH), 7.37 (m, 1H, Ar-CH), 7.40 (m, 1H, Ar-CH), 7.45 (m, 1H, Ar-CH), 7.48 (m, 1H, Ar-CH), 7.70 (m, 1H, *J* = 8.5 Hz, *J* = 2.5 Hz, Ar-CH), 7.71 (bs, 1H, NH_2_), 7.74 (d, 1H, *J =* 2.5, Ar-CH), 10.39 (s, 1H, COH).

*2′-bromo-4-methoxy-[1,1′-biphenyl]-3-carbaldehyde* (**IIIc**). *R*_f_ = 0.43 (hexane:ethyl acetate, 4:1); HPLC: 94.15%; m.p. 106 °C–107 °C; yield: 23.36%. ^1^H-NMR (500 MHz, DMSO): δ 3.98 (s, 3H, OCH_3_), 7.32 (m, 1H, *J* = 8 Hz, Ar-CH), 7.33 (d, 1H, *J* = 8.5 Hz, Ar-CH), 7.40 (m, 1H, *J* = 8 Hz, *J* = 2 Hz, *J* = 6 Hz, Ar-CH), 7.47 (m, 1H, *J* = 2 Hz, Ar-CH), 7.69 (d, 1H, *J =* 2, Ar-CH), 7.71 (dd, 1H, *J* = 8.5 Hz, *J* = 2 Hz, Ar-CH), 7.74 (m, 1H, Ar-CH), 10.39 (s, 1H, COH). ^13^C-NMR (100 MHz, DMSO): δ 56.2 (1C, OCH_3_), 112.7 (1C, phenyl), 121.8 (1C, phenyl), 123.6 (1C, phenyl), 128.2 (1C, phenyl), 128.3 (1C, phenyl),129.6 (1C, phenyl), 131.4 (1C, phenyl), 132.7 (2C, phenyl), 133.1 (1C, phenyl), 140.4 (1C, phenyl), 161.0 (1C, phenyl), 188.9 (1C, COH).

*4′-fluoro-4-methoxy-[1,1′-biphenyl]-3-carbaldehyde* (**IIId**). *R*_f_ = 0.41 (hexane:ethyl acetate, 2,5:1); HPLC: 99.038%; m.p. 81 °C; yield: 88.60%. ^1^H-NMR (500 MHz, DMSO): δ 3.96 (s, 3H, OCH_3_), 7.28 (m, 2H, *J* = 9 Hz, Ar-CH), 7.33 (d, 1H, *J* = 8.5 Hz, Ar-CH), 7.70 (m, 2H, Ar-CH), 7.90 (d, 1H, *J* = 2.5 Hz, Ar-CH), 7.96 (dd, 1H, *J* = 8.5 Hz, *J* = 2.5 Hz, Ar-CH), 10.39 (s, 1H, CHO). ^13^C-NMR (100 MHz, DMSO): δ 56.2 (1C, OCH_3_), 113.5 (1C, phenyl), 115.8 (2C, phenyl), 124.3 (1C, phenyl), 125.6 (1C, phenyl), 128.3 (2C, phenyl), 131.6 (1C, phenyl), 134.4 (1C, phenyl), 135.2 (1C, phenyl), 160.9 (1C, phenyl), 162.7 (1C, phenyl), 189.1 (1C, CHO).

*2-((4-methoxy-[1,1′-biphenyl]-3-yl)methylene)hydrazine-1-carbothioamide* (**IVa**). 5-phenyl-2-methoxybenzaldehyde (**IIIa**) and thiosemicarbazide in equimolar amounts were introduced in a three-necked flask and dissolved with *n*-propanol at 50 °C. Then 5 drops of acetic acid were added to the reaction mixture as a catalyst. After a few minutes the formation of an abundant white precipitate is observed (this indicates the formation of the thiosemicarbazone). The reaction was monitored by TLC (hexane:ethyl acetate, 1:1). After 6 hours the reaction was completed and the solid filtered. The compound is a white fluffy solid: *R*_f_ = 0.71 (hexane:ethyl acetate 1:1); HPLC: 95.933%; m.p. 223.3–223.8 °C; yield: 97%. ^1^H-NMR (500 MHz, DMSO): δ 3.87 (s, 3H, OCH_3_), 7.15 (d, 1H, *J* = 8.5 Hz, Ar-CH), 7.32 (m, 1H, Ar-CH), 7.44 (m, 2H, Ar-CH), 7.68 (dd, 1H, *J* = 8.5 Hz, Ar-CH), 7.72 (m, 2H, *J* = 8.5 Hz, Ar-CH) 8.18–8.19 (brs, 2H, NH_2_), 8.37 (d, 1H, Ar-CH), 8.45 (s, 1H, CH=N), 11.45 (s, 1H, NH). ^13^C-NMR (100 MHz, DMSO): δ 55.9 (1C, OCH_3_), 112.2 (1C, phenyl), 122.5 (1C, phenyl), 123,8 (1C, phenyl), 126.5 (2C, phenyl), 126.9 (1C, phenyl), 128.7 (2C, phenyl), 129.4 (1C, phenyl), 132.7 (1C, phenyl), 137.8 (1C, CH=N), 139.4 (1C, phenyl), 157.3 (1C, phenyl), 177.8 (1C, NH-CSNH_2_).

According to the above described procedure, the following compounds have been prepared:

*3′-[[(carbamothioylamino)imino]methyl]-4′-methoxy-[1,1′-biphenyl]-2-carboxamide* (**IVb**). *R*_f_ = 0.26 (hexane:ethyl acetate, 1:4); HPLC: 88.22%; m.p. 234–235 °C; yield: 82.48%. ^1^H-NMR (500 MHz, DMSO): δ 3.86 (s, 3H, OCH_3_), 7.09 (d, 1H, *J* = 8.5 Hz, Ar-CH), 7.33 (bs, 1H, NH_2_), 7.40 (dd, 1H, Ar-CH), 7.40 (m, 1H, Ar-CH), 7.41 (m, 2H, Ar-CH), 7.46 (m, 1H, Ar-CH), 7.66 (bs, 1H, NH_2_), 7.88 (bs, 1H, NH_2_), 8.15 (d, 1H, *J* = 2.5 Hz), 8.18 (bs, 1H, NH_2_), 8.43 (s, 1H, CH=N), 11.46 (s, 1H, NH-CSNH_2_). ^13^C-NMR (100 MHz, DMSO): δ 55.8 (1C, OCH_3_), 111.4 (1C, phenyl), 121.9 (1C, phenyl), 125.8 (1C, phenyl), 126.7 (1C, phenyl), 127.3 (1C, phenyl), 129.0 (1C, phenyl), 130.0 (1C, phenyl), 131.2 (2C, phenyl), 137.5 (1C, phenyl), 137.8 (1C, CH=N), 138.2 (1C, phenyl),157.1 (1C, phenyl),171.3 (1C, CONH_2_), 177.8 (1C, NH-CSNH_2_).

*(2-methoxy-5-(2-bromo)phenylphenyl)methylidene]amino] thiourea* (**IVc**). *R*_f_ = 0.725 (hexane:ethyl acetate, 1:1); HPLC: 93.98%; m.p. 219–220 °C; yield: 62.45%. ^1^H-NMR (500 MHz, DMSO): δ 3.88 (s, 3H, OCH_3_), 7.13 (d, 1H, *J* = 9 Hz), 7.39 (m, 1H, *J* = 1.5 Hz), 7.39 (m, 2H), 7.45 (m, 1H, *J* = 1 Hz), 7.72 (m, 1H, *J* = 8 Hz, *J* = 1.5 Hz), 8.10–8.06 (bs, 2H, NH), 8.14 (d, 1H, *J* = 2 Hz), 8.44 (s, 1H, CH=N), 11.43 (s, 1H, NH). ^13^C-NMR (100 MHz, DMSO): δ 55.9 (1C, OCH_3_), 111.2 (1C, phenyl), 121.9 (1C, phenyl), 122.2 (1C, phenyl), 126.6 (1C, phenyl), 127.8 (1C, phenyl), 129.1 (1C, phenyl), 131.6 (1C, phenyl), 132.0 (1C, phenyl), 132.8 (1C, phenyl), 133.2 (1C, phenyl), 137.5 (1C, CH=N), 141.4 (1C, phenyl), 157.2 (1C, phenyl), 177.8 (1C, NH-CSNH_2_).

*[{[5-(4-fluorophenyl)-2-methoxyphenyl]methylidene} amino] thiourea* (**IVd**). *R*_f_ = 0.63 (hexane:ethyl acetate, 1:1); HPLC: 99.98%; m.p. 217–218 °C; yield: 84%. NMR: ^1^H-NMR (500 MHz, DMSO): δ 3.87 (s, 3H, OCH_3_), 7.14 (d, 1H, *J =* 9, Ar-CH), 7.26 (m, 2H, *J* = 8.5 Hz, Ar-CH), 7.67 (dd, 1H, *J* = 8.5 Hz, *J* = 2 Hz, Ar-CH), 7.76 (m, 2H, *J* = 8.5 Hz, *J* = 2 Hz, Ar-CH), 8.21–8.18 (brd, 2H, NH_2_), 8.35 (d, 1H, *J* = 2.5 Hz, Ar-CH), 8.44 (s, 1H, CHN), 11.46 (s, 1H, NH). ^13^C-NMR (100 MHz, DMSO): δ 55.9 (1C, OCH_3_), 112.2 (1C, phenyl), 115.4 (1C, phenyl), 115.5 (1C, phenyl), 122.5 (1C, phenyl), 123.7 (1C, phenyl), 128.4 (1C, phenyl), 128.5 (1C, phenyl), 129.3 (1C, phenyl), 131.7 (1C, phenyl), 135.8 (1C, phenyl), 137.6 (1C, CH=N), 157.3 (1C, phenyl), 161.6 (1C, phenyl), 177.8 (1C, NH-CSNH_2_).

##### Preparation of 2-[2-[(2-Methoxy-5-Phenylphenyl)methylidene]hydrazin-1-yl]-4-(4-Methoxyphenyl)-1,3-Thiazole Bromide (**EMAC2056**)

2-((4-Methoxy-[1,1′-biphenyl]-3-yl)methylene)hydrazine-1-carbothioamide (**IVa**) (1 eq) was introduced in a flask and dissolved in ethanol. To this solution phenacyl bromide (1 eq) was added and the mixture stirred at r.t.. The reaction was monitored by TLC (hexane : ethyl acetate, 1:1). When completion of the reaction was reached, the obtained white solid was filtered. The desired compound was a white crystalline solid: *R*_f_ = 0.59 (hexane : ethyl acetate, 1:1); m.p. 209 °C decomposition; yield: 81.46%. ^1^H-NMR (500 MHz, DMSO): δ 3.78 (s, 3H, OCH_3_), 3.90 (s, 3H, OCH_3_), 6.97 (m, 2H, *J =* 9), 7.15 (s, 1H, CH thiazole), 7.19 (d, 1H, *J =* 9), 7.36 (m, 1H), 7.48 (m, 2H), 7.62 (m, 2H, *J =* 7.5), 7.67 (dd, 1H, *J =* 8.5, *J =* 2.5), 7.78 (m, 2H, *J =* 9), 8.03 (d, 1H, *J =* 2.5), 8.42 (s, 1H, CH=N). ^13^C-NMR (100 MHz, DMSO): δ 55.2 (1C, OCH_3_), 55.9 (1C, OCH_3_), 101.7 (1C, thiazole), 112.5 (1C, phenyl), 114.0 (2C, phenyl), 122.6 (1C, phenyl), 122.8 (1C, phenyl), 126.2 (2C, phenyl), 127.0 (2C, phenyl), 127.1 (1C, phenyl), 128.7 (1C, phenyl), 129.1 (2C, phenyl), 132.8 (1C, phenyl), 137.2 (1C, CH=N), 139.4 (1C, phenyl), 139.6 (1C, phenyl), 149.4 (1C, thiazole), 156.7 (1C, phenyl), 158.9 (1C, phenyl), 168.2 (1C, thiazole).

According to the above described procedure the following compounds were synthesized:

*4-(3,4-dichlorophenyl)-2-[2-[(2-methoxy-5-phenylphenyl)methylidene]hydrazin-1-yl]-1,3-thiazole bromide* (**EMAC2057**). *R*_f_ = 0.79 (hexane : ethyl acetate, 1:1); m.p. 213 °C decomposition; yield: 92.08%. ^1^H-NMR (500 MHz, DMSO): δ 3.90 (s, 3H, OCH3), 7.19 (d, 1H, *J* = 8.5 Hz, Ar-CH), 7.35 (m, 2H, Ar-CH), 7.48 (m, 2H, Ar-CH), 7.55 (s, 1H, CH thiazole), 7.62 (m, 1H, Ar-CH), 7.66 (d, 1H, *J* = 8.5 Hz), 7.67 (d, 1H, 8.5), 7.84 (dd, 1H, *J* = 8.5 Hz, *J* = 2 Hz), 8.01 (d, 1H, *J* = 2.5 Hz), 8.08 (d, 1H, *J* = 2 Hz), 8.40 (s, 1H, CH=N), 12.2 (brs, 1H, NH). ^13^C-NMR (100 MHz, DMSO): δ 55.9 (1C, OCH3), 106.0 (1C, thiazole), 112.5 (1C, phenyl), 122.6 (1C, phenyl), 122.7 (1C, phenyl), 125.6 (1C, phenyl), 126.2 (2C, phenyl), 127.1 (1C, phenyl), 127.2 (1C, phenyl), 129.0 (2C, phenyl), 129.1 (1C, phenyl), 129.7 (1C, phenyl), 130.9 (1C, phenyl), 131.4 (1C, phenyl), 132.8 (1C, phenyl), 135.2 (1C, phenyl), 136.9 (1C, CH=N), 139.6 (1C, phenyl), 147.9 (1C, thiazole), 156.7 (1C, phenyl), 168.5 (1C, thiazole).

*4-(3-chlorophenyl)-2-[2-[(2-methoxy-5-phenylphenyl)methylidene]hydrazin-1-yl]-1,3-thiazole bromide* (**EMAC2058**). *R*_f_ = 0.77 (hexane : ethyl acetate, 1:1); m.p. 215 °C decomposition; yield: 91.27%. ^1^H-NMR (500 MHz, DMSO): δ 3.90 (s, 3H, OCH_3_), 7.19 (d, 1H, *J* = 8.5 Hz, Ar-CH), 7.36 (m, 1H, Ar-CH), 7.39 (s, 1H, CH thiazole), 7.46 (m, 2H, Ar-CH), 7.48 (m, 2H, Ar-CH), 7.62 (d, 2H, *J* = 7.5 Hz, Ar-CH), 7.67 (dd, 1H, *J* = 8.5 Hz, *J* = 2.5 Hz, Ar-CH), 7.87 (d, 2H, *J* = 8.5 Hz, Ar-CH), 8.02 (d, 1H, *J* = 2.5 Hz, Ar-CH), 8.40 (s, 1H, CH=N, Ar-CH), 12.24 (brs, 1H, NH, Ar-CH). ^13^C-NMR (100 MHz, DMSO): δ 55.9 (1C, OCH_3_), 101.6 (1C, thiazole), 111.7 (1C, phenyl), 122.7 (1C, phenyl), 122.7 (1C, phenyl), 126.3 (2C, phenyl), 127.1 (1C, phenyl), 127.3 (2C, phenyl), 128.6 (2C, phenyl), 129.0 (2C, phenyl), 129.1 (1C, phenyl), 131.9 (1C, phenyl), 132.8 (1C, phenyl), 133.4 (1C, phenyl), 136.8 (1C, CH=N), 139.6 (1C, phenyl), 149,1 (1C, thiazole), 156.7 (1C, phenyl), 168.4 (1C, thiazole).

*4-(4-nitrophenyl)-2-[2-[(2-methoxy-5-phenylphenyl)methylidene]hydrazin-1-yl]-1,3-thiazole bromide* (**EMAC2059**). *R*_f_ = 0.69 (hexane : ethyl acetate, 1:1); m.p. 208 °C decomposition; yield: 89.39%. ^1^H-NMR (500 MHz, DMSO): δ 3.90 (s, 3H, OCH_3_), 7.19 (d, 1H, *J* = 8.5 Hz, Ar-CH), 7.36 (m, 1H, Ar-CH), 7.48 (m, 2H, Ar-CH), 7.63 (d, 2H, *J* = 7.5 Hz, Ar-CH), 7.67 (dd, 1H, *J* = 8.5 Hz, *J* = 2.5 Hz, Ar-CH), 7.71 (s, 1H, thiazole), 8.02 (d, 1H, *J* = 2.5 Hz, Ar-CH), 8.11 (d, 2H, *J =* 9, Ar-CH), 8.27 (d, 2H, *J =* 9, Ar-CH), 8.41 (s, 1H, CH=N), 12.32 (brs, 1H, NH). ^13^C-NMR (100 MHz, DMSO): δ 55.9 (1C, OCH_3_), 108.6 (1C, thiazole), 112.5 (1C, phenyl), 122.6 (1C, phenyl), 122.8 (1C, phenyl), 124.1 (2C, phenyl), 126.3 (2C, phenyl), 126.3 (2C, phenyl), 127.1 (1C, phenyl), 129.0 (2C, phenyl), 129.2 (1C, phenyl), 132.8 (1C, phenyl), 137.1 (1C, CH=N), 139.6 (1C, phenyl), 140.6 (1C, phenyl), 146.2 (1C, phenyl), 148.5 (1C, thiazole), 156.7 (1C, phenyl), 168.7 (1C, thiazole).

*2-{4-methoxy-3-[(1E)-{2-[4-(4-methoxyphenyl)-1,3-thiazol-2-yl]hydrazin-1-ylidene}methyl] phenyl}benzamide bromide* (**EMAC2060**). *R*_f_ = 0.31 (hexane : ethyl acetate, 1:4); m.p. 203 °C decomposition; yield: 70.5%. ^1^H-NMR (500 MHz, DMSO): δ 3.78 (s, 3H, OCH_3_), 3.90 (s, 3H, OCH_3_), 6.50 (bs, 1H, CONH_2_), 6.96 (d, 2H, *J* = 9 Hz, Ar-CH), 7.13 (m, 2H, thiazole + Ar-CH), 7.30–7.50 (m, 8H, Ar-CH), 7.72 (brs, 1H, CONH_2_), 7.86 (d, 1H, *J* = 2.5 Hz), 12.19 (brs, 1H, NH). ^13^C-NMR (100 MHz, DMSO): δ 55.2 (1C, OCH_3_), 55.8 (1C, OCH_3_), 106.0 (1C, thiazole), 111.6 (1C, phenyl), 122.0 (1C, phenyl), 124.8 (1C, phenyl), 126.8 (1C, phenyl), 127.3 (2C, phenyl), 127.6 (1C, phenyl), 128.6 (2C, phenyl), 129.3 (1C, phenyl), 129.7 (1C, phenyl), 130.7 (1C, phenyl), 131.9 (1C, phenyl), 133.1 (1C, phenyl), 133.4 (1C, phenyl), 136.9 (1C, CH=N), 137.3 (1C, phenyl), 138.3 (1C, phenyl), 149.1 (1C, thiazole), 156.4 (1C, phenyl), 168.4 (1C, thiazole), 171.1 (1C, CONH_2_).

*2-{3-[{2-[4-(3,4-dichlorophenyl)-1,3-thiazol-2-yl]hydrazin-1-ylidene}methyl]-4-methoxyphenyl}benzamide bromide* (**EMAC2061**). *R*_f_ = 0.41 (hexane : ethyl acetate, 1:4); m.p. 221 °C decomposition; yield: 77.71%. ^1^H-NMR (500 MHz, DMSO): δ 3.89 (s, 3H, OCH_3_), 7.13 (d, 1H, *J* = 9 Hz, Ar-CH), 7.32 (bs, 1H, NH_2_), 7.37 (m, 1H, Ar-CH), 7.39 (m, 1H, Ar-CH), 7.41 (m, 1H, Ar-CH), 7.49 (m, 2H, Ar-CH), 7.53 (s, 1H, thiazole), 7.66 (d, 1H, *J* = 8.5 Hz, Ar-CH), 7.72 (brs, 1H, NH_2_), 7.83 (dd, 1H, *J =* 8.5, *J* = 2 Hz, Ar-CH), 7.87 (d, 1H, *J* = 2.5 Hz, Ar-CH), 8.08 (d, 1H, *J* = 2 Hz, Ar-CH), 8.38 (s, 1H, CH=N), 12.21 (brs, 1H, NH). ^13^C-NMR (100 MHz, DMSO): δ 55.8 (1C, OCH_3_), 106.0 (1C, thiazole), 111.6 (1C, phenyl), 122.0 (1C, phenyl), 124.8 (1C, phenyl), 125.6 (1C, phenyl), 126.8 (1C, phenyl), 127.1 (1C, phenyl), 127.6 (1C, phenyl), 129.3 (1C, phenyl), 129.6 (1C, phenyl), 129.7 (1C, phenyl), 130.7 (1C, phenyl), 130.9 (1C, phenyl), 131.4 (1C, phenyl), 133.1 (1C, phenyl), 135.2 (1C, phenyl), 136.9 (1C, CH=N), 137.3 (1C, phenyl), 138.3 (1C, phenyl), 147.9 (1C, thiazole), 156.4 (1C, phenyl), 168.5 (1C, thiazole), 171.1 (1C, CONH_2_).

*2-{3-[{2-[4-(4-chlorophenyl)-1,3-thiazol-2-yl]hydrazin-1-ylidene}methyl]-4-methoxyphenyl}benzamide bromide* (**EMAC2062**). *R*_f_ = 0.38 (hexane : ethyl acetate, 1:4); m.p. 222 °C decomposition; yield: 84.74%. ^1^H-NMR (500 MHz, DMSO): δ 3.89 (s,3H, OCH_3_), 7.13 (d, 1H, *J* = 9 Hz, Ar-CH), 7.32 (bs, 1H, CONH_2_), 7.37 (s, 1H, thiazole), 7.37 (m, 1H, Ar-CH), 7.40 (m, 1H, Ar-CH), 7.41 (dd, 1H, Ar-CH), 7.44 (m, 1H, Ar-CH), 7.46 (m, 2H, Ar-CH), 7.49 (m, 2H, Ar-CH), 7.72 (brs, 1H, CONH_2_), 7.86 (m, 2H, Ar-CH), 7.87 (s, 1H, Ar-CH), 12.19 (bs, 1H, NH). ^13^C-NMR (100 MHz, DMSO): δ 55.8 (1C, OCH_3_), 106.0 (1C, thiazole), 111.6 (1C, phenyl), 122.0 (1C, phenyl), 124.8 (1C, phenyl), 126.8 (1C, phenyl), 127.3 (2C, phenyl), 127.6 (1C, phenyl), 128.6 (2C, phenyl), 129.3 (1C, phenyl), 129.7 (1C, phenyl), 130.7 (1C, phenyl), 131.9 (1C, phenyl), 133.1 (1C, phenyl), 133.4 (1C, phenyl), 136.9 (1C, CH=N), 137.3 (1C, phenyl), 138.3 (1C, phenyl), 149.1 (1C, thiazole), 156.4 (1C, phenyl), 168.4 (1C, thiazole), 171.1 (1C, CONH_2_).

*2-{3-[(2-{4-[4-nitrophenyl]-1,3-thiazol-2-yl}hydrazin-1-ylidene)methyl]-4-methoxyphenyl}benzamide bromide* (**EMAC2063**). *R*_f_ = 0.33 (hexane : ethyl acetate, 1:4); m.p. 208 °C decomposition; yield: 89.39%. ^1^H-NMR (500 MHz, DMSO): δ 3.89 (s,3H, OCH_3_), 7.13 (d, 1H, *J* = 9 Hz, Ar-CH), 7.32 (bs, 1H, CONH_2_), 7.38 (m, 1H, Ar-CH), 7.40 (m, 1H, Ar-CH), 7.41 (dd, 1H, Ar-CH), 7.44 (m, 1H, Ar-CH), 7.49 (m, 1H, Ar-CH), 7.70 (s, 1H, thiazole), 7.73 (brs, 1H, CONH_2_), 7.88 (d, 1H, *J* = 2.5 Hz, Ar-CH), 8.11 (m, 2H, *J* = 9 Hz, Ar-CH), 8.27 (m, 2H, *J* = 8.5 Hz, Ar-CH), 8.39 (s, 1H, CH=N), 12.28 (bs, 1H, NH). ^13^C-NMR (100 MHz, DMSO): δ 55.9 (1C, OCH_3_), 108.6 (1C, thiazole), 111.7 (1C, phenyl), 121.9 (1C, phenyl), 124.1 (2C, phenyl), 124.8 (1C, phenyl), 126.3 (2C, phenyl), 126.8 (1C, phenyl), 127.6 (1C, phenyl), 129.3 (1C, phenyl), 129.7 (1C, phenyl), 130.8 (1C, phenyl), 133.1 (1C, phenyl), 137.1 (1C, CH=N), 137.3 (1C, phenyl), 138.3 (1C, phenyl), 140.7 (1C, phenyl), 146.2 (1C, phenyl), 148.5 (1C, thiazole), 156.5 (1C, phenyl), 168.7 (1C, thiazole), 171.1 (1C, CONH_2_).

*2-{[5-(2-bromophenyl)-2-methoxyphenyl]methylidene}hydrazin-1-yl]-4-(4-methoxyphenyl)-1,3-thiazole bromide* (**EMAC2064**). *R*_f_ = 0.70 (hexane : ethyl acetate, 1:1); m.p. 193 °C decomposition; yield: 83.65%. ^1^H-NMR (500 MHz, DMSO): δ 3.78 (s, 3H, OCH_3_), 3.90 (s, 3H, OCH_3_), 6.97 (m, 2H, *J* = 8.5 Hz, Ar-CH), 7.16 (s, 1H, thiazole), 7.18 (d, 2H, *J* = 8.5, Ar-CH), 7.30 (m, 2H, Ar-CH), 7.64 (dd, 2H, *J* = 8.5 Hz, *J* = 2.5 Hz, Ar-CH), 7.77 (m, 2H, *J* = 8.5 Hz, Ar-CH), 7.98 (d, 1H, *J* = 2 Hz, Ar-CH), 8.41 (s, 1H, CH=N), 12.28 (brs, 1H, NH). ^13^C-NMR (100 MHz, DMSO): δ 55.2 (1C, OCH_3_), 55.9 (1C, OCH_3_), 101.7 (1C, thiazole), 112.5 (1C, phenyl), 114.0 (2C, phenyl), 115.7 (1C, phenyl), 115.9 (1C, phenyl), 122.7 (1C, phenyl), 122.7 (1C, phenyl), 126.9 (1C, phenyl), 127.0 (2C, phenyl), 128.2 (1C, phenyl), 128.3 (1C, phenyl), 129.1 (1C, phenyl), 131.8 (1C, phenyl), 136.1 (1C, phenyl), 137.1 (1C, CH=N), 149.4 (1C, thiazole), 156.7 (1C, phenyl), 158.9 (1C, phenyl), 161.6 (1C, phenyl), 168.2 (1C, thiazole).

*2-{[5-(2-bromophenyl)-2-methoxyphenyl]methylidene}hydrazin-1-yl]-4-(3,4-dicholophenyl)-1,3-thiazole bromide* (**EMAC2065**). *R*_f_ = 0.86 (hexane : ethyl acetate, 1:1); m.p. 220 °C decomposition; yield: 91.36%. ^1^H-NMR (500 MHz, DMSO): δ 3.91 (s, 3H, OCH_3_), 7.18 (d, 1H, *J* = 9 Hz), 7.32 (m, 1H), 7.40 (dd, 1H, *J* = 9 Hz, *J* = 2.5 Hz), 7.42 (m, 1H, *J* = 7.5 Hz, *J* = 2 Hz), 7.47 (m, 1H, *J* = 1 Hz) 7.51 (s, 1H thiazole), 7,56 (d,1H, *J* = 8.5 Hz), 7.75 (m, 1H, *J* = 6.5 Hz, *J* = 1.5 Hz), 7.80 (d, 1H, *J* = 2.5 Hz), 7.82 (dd, 1H, *J* = 6.5 Hz, *J* = 2 Hz), 8.07 (d, 1H, *J* = 2 Hz), 8.39 (s, 1H, CH=N), 12.24 (bs, 1H, NH). ^13^C-NMR (100 MHz, MHz, DMSO): δ 55.9 (1C, OCH_3_), 106.0 (1C, thiazole), 111.7 (1C, phenyl), 121.9 (1C, phenyl), 122.0 (1C, phenyl), 125.5 (1C, phenyl), 125.6 (1C, phenyl), 127.1 (1C, phenyl), 128.1 (1C, phenyl), 129.3 (1C, phenyl), 129.7 (1C, phenyl), 130.9 (1C, phenyl), 131.4 (1C, phenyl), 131.4 (1C, phenyl), 131.5 (1C, phenyl), 132.9 (1C, phenyl),133.1 (1C, phenyl), 135.2 (1C, phenyl), 136.7 (1C, CH=N), 141.2 (1C, phenyl), 147.9 (1C, thiazole), 156.6 (1C, phenyl), 168.4 (1C, thiazole).

*2-{[5-(2-bromophenyl)-2-methoxyphenyl]methylidene}hydrazin-1-yl]-4-(4-cholophenyl)-1,3-thiazole bromide* (**EMAC2066**). *R*_f_ = 0.82 (hexane : ethyl acetate, 1:1); m.p. 218 °C decomposition; yield: 98%. ^1^H-NMR (500 MHz, DMSO): δ 3.91 (s, 3H, OCH_3_), 7.18 (d, 1H, *J* = 8.5 Hz), 7.32 (m, 1H), 7.35 (s, 1H, thiazole), 7.40 (dd, 1H, *J* = 8.5 Hz, *J* = 2.5 Hz), 7.42 (m, 1H), 7.45 (m, 2H, *J* = 8.5 Hz), 7.48 (m, 1H, *J* = 1.5 Hz), 7.75 (m, 1H, *J* = 8 Hz, *J* = 2 Hz), 7.8 (d, 1H, *J* = 2 Hz), 7.85 (m, 2H, *J* = 8.5 Hz, *J* = 2 Hz), 8.4 (s, 1H, CH=N), 12.21 (bs, 1H, NH). ^13^C-NMR (100 MHz, DMSO): δ 55.9 (1C, OCH_3_), 104.5 (1C, thiazole), 111.7 (1C, phenyl), 121.9 (1C, phenyl), 122.0 (1C, phenyl), 125.5 (1C, phenyl), 127.2 (2C, phenyl), 128.1 (1C, phenyl), 128.6 (2C, phenyl), 129.3 (1C, phenyl), 131.4 (1C, phenyl),131.5 (1C, phenyl), 131.9 (1C, phenyl), 131.9 (1C, phenyl), 133.1 (1C, phenyl), 133.4 (1C, phenyl), 136.6 (1C, CH=N), 141.2 (1C, phenyl), 149.1 (1C, thiazole), 156.6 (1C, phenyl), 168.3 (1C, thiazole).

*2-{[5-(2-bromophenyl)-2-methoxyphenyl]methylidene}hydrazin-1-yl]-4-(4-nitrophenyl)-1,3-thiazole bromide* (**EMAC2067**). *R*_f_ = 0.81 (hexane : ethyl acetate, 1:1); m.p. 233 °C decomposition; yield: 91.2%. ^1^H-NMR (500 MHz, DMSO): δ 3.91 (s, 3H, OCH_3_), 7.18 (d, 1H, *J* = 8.5 Hz), 7.32 (m, 1H), 7.40 (dd, 1H, *J* = 8.5 Hz, *J* = 2.5 Hz), 7.42 (m, 1H), 7.48 (m, 1H), 7.68 (s, 1H, thiazole), 7.75. (m, 1H, *J* = 8 Hz, *J* = 1 Hz), 7.81 (d, 1H, *J* = 2.5 Hz), 8.10 (m, 2H, *J* = 9 Hz), 8.26 (m, 2H, *J* = 8.5 Hz), 8.40 (s, 1H, CH=N), 12.32 (bs, 1H, NH). ^13^C-NMR (100 MHz, DMSO): δ 55.9 (1C, OCH_3_), 108.6 (1C, thiazole), 111.7 (1C, phenyl), 121.9 (1C, phenyl), 122.0 (1C, phenyl), 124.1 (2C, phenyl), 125.5 (1C, phenyl), 126.3 (2C, phenyl), 128.1 (1C, phenyl), 129.3 (1C, phenyl), 131.4 (1C, phenyl), 131.6 (1C, phenyl), 132.9 (1C, phenyl), 133.1 (1C, phenyl), 136.8 (1C, CH=N), 140.6 (1C, phenyl), 141.2 (1C, phenyl), 146.2 (1C, phenyl), 148.5 (1C, thiazole), 156.6 (1C, phenyl), 168.6 (1C, thiazole).

*2-[2-{[5-(4-fluorophenyl)-2-methoxyphenyl]methylidene}hydrazin-1-yl]-4-(4-methoxyphenyl)-1,3-thiazole bromide* (**EMAC2068**). *R*_f_ = 0.775 (hexane : ethyl acetate, 1:1); m.p. 186 °C decomposition; yield: 82.28%. ^1^H-NMR (500 MHz, DMSO): δ 3.78 (s, 3H, OCH_3_), 3.90 (s, 3H, OCH_3_), 6.97 (m, 2H, *J* = 8.5 Hz), 7.16 (s, 1H, thiazole), 7.18 (d, 1H, *J* = 8.5 Hz), 7.30 (m, 2H), 7.64 (dd, 1H, *J* = 8.5 Hz, *J* = 2.5 Hz), 7.77 (m, 2H, *J* = 8.5 Hz), 7.98 (d, 1H, *J* = 2 Hz), 8.41 (s, 1H, CH=N), 12.28 (brs, 1H, NH). ^13^C-NMR (100 MHz, DMSO): δ 55.2 (1C, OCH_3_), 55.9 (1C, OCH_3_), 101.7 (1C, thiazole), 112.5 (1C, phenyl), 114.0 (2C, phenyl), 115.8 (2C oF, phenyl, *J* = 21 Hz), 122.7 (1C, phenyl), 122.8 (1C, phenyl), 126.9 (1C, phenyl), 127.0 (2C, phenyl), 128.2 (2C, phenyl), 129.1 (1C, phenyl), 131.8 (1C, phenyl), 136.1 (1C, phenyl), 137.1 (1C, CH=N), 149.4 (1C, thiazole), 156.7 (1C, phenyl), 158.9 (1C, phenyl), 161.6 (1C-F, phenyl, *J* = 245 Hz), 168.2 (1C, thiazole).

*4-(3,4-dichlorophenyl)-2-[-2-{[5-(4-fluorophenyl)-2-methoxyphenyl]methylidene}hydrazin-1-yl]-1,3-thiazole bromide* (**EMAC2069**). *R*_f_ = 0.91 (hexane : ethyl acetate, 1:1); m.p. 225 °C decomposition; yield: 87.75%. ^1^H-NMR (500 MHz, DMSO): δ 3.90 (s, 3H, OCH_3_), 7.18 (d,1H, *J* = 9 Hz), 7.30 (m, 2H), 7.55 (s, 1H, thiazole), 7.64 (dd, 1H), 7.65 (m, 2H), 7.66 (d, 1H), 7.83 (dd, 1H, *J* = 8.5 Hz, *J* = 2 Hz), 7.97 (d, 1H, *J* = 2 Hz), 8.08 (d, 1H, *J* = 2 Hz), 8.39 (s, 1H, CHN), 12.26 (brs, 1H, NH). ^13^C-NMR (100 MHz, DMSO): δ 55.9 (1C, OCH_3_), 106.0 (1C, thiazole), 112.5 (1C, phenyl), 115.8 (2C oF, phenyl, *J* = 21 Hz), 122.6 (1C, phenyl), 122.7 (1C, phenyl), 125.6 (1C, phenyl), 127.2 (1C, phenyl), 128.2 (1C, phenyl), 128.3 (1C, phenyl), 129.7 (2C, phenyl), 130.9 (1C, phenyl), 131.4 (1C, phenyl), 131.8 (1C, phenyl), 135.2 (1C, phenyl), 136.1 (1C, phenyl), 136.9 (1C, CH=N), 147.9 (1C, thiazole), 156.7 (1C, phenyl), 161.6 (1C-F, phenyl, *J* = 245 Hz), 168.5 (1C, thiazole).

*4-(4-chlorophenyl)-2-[-2-{[5-(4-fluorophenyl)-2-methoxyphenyl]methylidene}hydrazin-1-yl]-1,3-thiazole bromide* (**EMAC2070**). *R*_f_ = 0.875 (hexane : ethyl acetate, 1:1); m.p. 235 °C decomposition; yield: 86.9%. ^1^H-NMR (500 MHz, DMSO): δ 3.90 (s, 3H, OCH_3_), 7.18 (d, 1H, *J* = 8.5 Hz), 7.30 (m, 2H, *J* = 8.5 Hz), 7.39 (s, 1H, thiazole), 7.46 (m, 2H, *J* = 9 Hz), 7.64 (dd, 1H), 7.65 (m, 2H), 7.87 (m, 2H, *J* = 8.5 Hz), 7.97 (d, 1H, *J* = 2 Hz), 8.40 (s, 1H, CHN), 12.25 (brs, 1H, NH). ^13^C-NMR (100 MHz, DMSO): δ 55.9 (1C, OCH_3_), 104.6 (1C, thiazole), 112.5 (1C, phenyl), 115.8 (2C oF, phenyl, *J* = 21 Hz), 122.7 (1C, phenyl), 127.3 (1C, phenyl), 128.2 (2C, phenyl), 128.3 (1C, phenyl), 128.6 (2C, phenyl), 129.1 (2C, phenyl), 131.8 (1C, phenyl), 132.0 (1C, phenyl), 133.4 (1C, phenyl), 136.1 (1C, phenyl), 136.8 (1C, CH=N), 149.1 (1C, thiazole), 156.7 (1C, phenyl), 161.6 (1C-F, phenyl, *J* = 245 Hz), 168.4 (1C, thiazole).

*[(4-{2-[2-{[5-(4-fluorophenyl)-2-methoxyphenyl]methylidene}hydrazin-1-yl]-1,3-thiazol-4-yl}phenyl)nitroso]oxidanol bromide* (**EMAC2071**). *R*_f_ = 0.85 (hexane : ethyl acetate, 1:1); m.p. 237 °C decomposition; yield: 93.93%. ^1^H-NMR (500 MHz, DMSO): δ 3.90 (s, 3H, OCH_3_), 7.18 (d, 1H, *J* = 8.5 Hz), 7.30 (m, 2H), 7.65 (m, 3H), 7.72 (s, 1H, thiazole), 7.97 (d, 1H, *J* = 2.5 Hz), 8.11 (m, 2H, *J* = 9 Hz), 8.27 (m, 2H, *J* = 9 Hz), 8.40 (s, 1H, CHN), 12.32 (brs, 1H, NH). ^13^C-NMR (100 MHz, DMSO): δ 55.9 (1C, OCH_3_), 108.7 (1C, thiazole), 112.5 (1C, phenyl), 115.8 (2C oF, phenyl, *J* = 21 Hz), 122.6 (1C, phenyl), 124.1 (2C, phenyl), 126.4 (2C, phenyl), 128.2 (1C, phenyl), 128.3 (1C, phenyl), 129.1 (2C, phenyl), 131.8 (1C, phenyl), 136.1 (1C, phenyl), 137.0 (1C, CH=N), 140.6 (1C, phenyl), 146.2 (1C, phenyl), 148.5 (1C, thiazole), 156.7 (1C, phenyl), 161.6 (1C-F, phenyl, *J* = 245 Hz), 168.6 (1C, thiazole).

### 3.2. Biological Evaluation

#### 3.2.1. HIV-1 RT-Associated DNA Polymerase-Independent RNase H Activity Determination

The full length HIV-1 RT group M subtype B was expressed and purified as previously described [38]. The HIV RT-associated RNase H activity was measured as described [53]. Briefly, the reaction was performed in in 100 μL volume containing 50 mM Tris HCl pH 7.8, 6 mM MgCl_2_, 1 mM dithiothreitol (DTT), 80 mM KCl, 250 nM hybrid RNA/DNA (50-GTTTTCTTTTCCCCCCTGAC-30-Fluorescein, 50-CAAAAG AAAAGGGGGGACUG-30-Dabcyl) reaction was started by the addition of 20 ng of HIV-1 wild type (wt) RT and incubated for 1h at 37 °C. Reactions were stopped by the addition of EDTA and products were measured with a Perkin–Elmer Victor 3 multilabel counter plate reader (Waltham, MA, USA) at excitation–emission wavelength of 490/528 nm (Table 1).

#### 3.2.2. HIV-1 RT-Associated RNA Dependent DNA Polymerase Activity Determination

The HIV-1 RT-associated RNA-Dependent DP activity was measured as previously described [54,55]. Briefly, 20 ng of HIV-1 wt RT were incubated for 30 min at 37 °C in 25 μL volume containing 60 mM Tris-HCl pH 8.1, 8 mM MgCl_2_, 60 mM KCl, 13 mM DTT, 2.5 mM poly(A)-oligo(dT), 100 mM dTTP. The enzymatic reaction was stopped by the addition of EDTA. Reaction products were detected by picogreen addition and measured with a Perkin–Elmer Victor 3 multilabel counter plate reader at excitation–emission wavelength of 502/523 nm.

### 3.3. Molecular Medelling

#### 3.3.1. Ligand Preparation

Theoretical 3D models of the compounds **EMAC2056**–**2071** were built utilizing Maestro GUI [20] and considering both E and Z configurations. Then, the ligands’ most stable conformation was obtained by molecular mechanics conformational analysis with Macromodel software version 9.2. (Schrodinger LLC, New York, NY, USA) [56], considering MMFFs [57] as force field and solvent effects by adopting the implicit solvation model Generalized Born/Surface Area (GB/SA) water [58]. The simulations were performed allowing 5000 steps Monte Carlo analysis with Polak–Ribier Conjugate Gradient (PRCG) method and a convergence criterion of 0.05 kcal/(mol·Å).

#### 3.3.2. Protein Preparation

The coordinates for RT enzymes were taken from the RCSB Protein Data Bank [20] (PDB codes 1vrt [47], 2zd1 [48], 1ep4 [49], 3qo9 [50], 1rti [47], 1tv6 [51], 3lp2 [52]). The proteins were prepared by using the Maestro Protein Preparation Wizard protocol _ENREF_20 [20] and discarding the water molecules co-crystallized.

#### 3.3.3. Docking Experiments

QM-Polarized Ligand Docking default settings were applied [59]. The docking grids were defined by centering on W229 and Q500. The grid boxes of the same size (45 × 45 × 45 Å) covered overall the whole p66 subunit. The best solutions were subjected to the post-docking procedure. To better take into account the induced fit phenomena, the most energy-favoured generated complexes were fully optimized with 5000 steps of the Polak–Ribier conjugate gradient (PRCG) minimization method using OPLS2005 force field. The optimization process was performed up to the derivative convergence criterion equal to 0.1 kJ/(mol·Å)^−1^.

#### 3.3.4. Figures

The resulting best complexes were considered for the binding modes graphical analysis with LigandScout (inte:Ligand, Vienna, Austria) [60,61], and Ligand Interaction module included in Maestro GUI [20].

## 4. Conclusions

A series of new biphenylhydrazo 4-arylthiazoles derivatives has been synthesized to evaluate the potential of these derivatives as HIV-RT dual associated functions inhibitors. Their activity was compared with the known NNRTi efavirenz and with the RNase H inhibitor β-thujaplicinol. Both reference compounds, efavirenz and β-thujaplicinol, were more potent with respect to the new EMAC derivatives, but were not able to perform a dual inhibition of the two associated RT functions, being either RDDP or RNase H inhibitors. On the contrary, although with some differences, all EMAC compounds were able to inhibit both RDDP and RNase H functions indicating that they might represent the starting point for the design of new and more efficient RT full activity inhibitors. Derivative **EMAC2063** was the most potent derivative with an IC_50_ of 4.5 and 8.0 μM towards RNase H and RDDP, respectively. Docking experiments highlighted the relevant role of the 2-amido substituent in stabilising the ligand/enzyme complex. All together these data prompted us towards further investigation to better characterize the mechanism of action and clearly define the binding mode of such derivatives in order to acquire more detailed information that will be used for the design of more efficient RT inhibitors.

## Figures and Tables

**Table 1:** Activity of compounds **EMAC2056**–**2071** on HIV-1 RT-associated enzymatic functions RNase H and RDDP.

Compound	R	R’	RNase H ^a^ IC_50_ (µM)	RDDP ^b^ IC_50_ (µM)
**EMAC2056**	H	4-OCH_3_	19.5 ± 5.5	36.0 ± 5.0
**EMAC2057**	H	3,4-Cl	57.0 ± 5.0	88.0 ± 12.0
**EMAC2058**	H	4-Cl	46.0 ± 1.0	56.5 ± 9.5
**EMAC2059**	H	4-NO_2_	12.8 ± 4.3	39.5 ± 11.5
**EMAC2060**	2-CONH_2_	4-OCH_3_	21.5 ± 3.5	27.0 ± 1.0
**EMAC2061**	2-CONH_2_	3,4-Cl	6.5 ± 2.5	19.5 ± 0.5
**EMAC2062**	2-CONH_2_	4-Cl	8.0 ± 2.0	17.0 ± 2.0
**EMAC2063**	2-CONH_2_	4-NO_2_	4.5 ± 0.5	8.0 ± 0.0
**EMAC2064**	2-Br	4-OCH_3_	13.0 ± 4.0	32.5 ± 2.5
**EMAC2065**	2-Br	3,4-Cl	15.0 ± 1.0	53.5 ± 15.5
**EMAC2066**	2-Br	4-Cl	47.0 ± 3.0	71.5 ± 6.5
**EMAC2067**	2-Br	4-NO_2_	27.0 ± 5.0	30.0 ± 3.0
**EMAC2068**	4-F	4-OCH_3_	29.5 ± 3.5	61.5 ± 8.5
**EMAC2069**	4-F	3,4-Cl	52.5 ± 10.5	66.0 ± 2.0
**EMAC2070**	4-F	4-Cl	55.5 ± 1.5	59.0 ± 6.0
**EMAC2071**	4-F	4-NO_2_	19.0 ± 1.0	47.5 ± 11.5
**Efavirenz**	//	//	*	0.023 ± 0.002
**β-Thujaplicinol**	//	//	0.19 ± 0.03	*

^a^ Compound concentration required to reduce the HIV-1 RT-associated RNase H activity by 50%. ^b^ Compound concentration required to reduce the HIV-1 RT-associated RNA-dependent DNA-polymerase activity by 50%. * Not tested; //—Not applicable.

## Data Availability

Data are available under request to the authors.

## Sample Availability

Samples of the compounds are available from the authors.

## References

[1] Gallo R.C., Montagnier L. (2003). The discovery of HIV as the cause of AIDS. N. Engl. J. Med..

[2] Shaw G.M., Broder S., Essex M., Gallo R.C. (1984). Human T-cell leukemia virus: Its discovery and role in leukemogenesis and immunosuppression. Adv. Intern. Med..

[3] Shridhar T.D. (2020). Antiretroviral therapy—The future of HIV treatment. World J. Pharm. Res..

[4] Saag M.S., Gandhi R.T., Hoy J.F., Landovitz R.J., Thompson M.A., Sax P.E., Smith D.M., Benson C.A., Buchbinder S.P., del Rio C. (2020). Antiretroviral drugs for treatment and prevention of HIV infection in adults: 2020 recommendations of the international antiviral society—USA panel. JAMA.

[5] Lal H., Kumar T., Dutta S., Ram K. (2020). A concise review of existing therapies and recent advances in the management of HIV infection. Int. J. Pharm. Sci. Rev. Res..

[6] Tseng A., Seet J., Phillips E.J. (2015). The evolution of three decades of antiretroviral therapy: Challenges, triumphs and the promise of the future. Br. J. Clin. Pharmacol..

[7] Fischl M.A., Richman D.D., Grieco M.H., Gottlieb M.S., Volberding P.A., Laskin O.L., Leedom J.M., Groopman J.E., Mildvan D., Schooley R.T. (1987). The efficacy of Azidothymidine (AZT) in the treatment of patients with AIDS and AIDS-related complex. N. Engl. J. Med..

[8] de Béthune M.-P. (2010). Non-Nucleoside Reverse Transcriptase Inhibitors (NNRTIs), their discovery, development, and use in the treatment of HIV-1 infection: A review of the last 20 years (1989–2009). Antivir. Res..

[9] Flexner C. (1998). HIV-protease inhibitors. N. Engl. J. Med..

[10] Carter N.J., Keating G.M. (2007). Maraviroc. Drugs.

[11] Beccari M.V., Mogle B.T., Sidman E.F., Mastro K.A., Asiago-Reddy E., Kufel W.D. (2019). Ibalizumab, a novel monoclonal antibody for the management of multidrug-resistant HIV-1 infection. Antimicrob. Agents Chemother..

[12] LaBonte J., Lebbos J., Kirkpatrick P. (2003). Enfuvirtide. Nat. Rev. Drug Discov..

[13] Rossetti B., Montagnani F., De Luca A. (2018). Current and emerging two-drug approaches for HIV-1 therapy in ART-naïve and ART-experienced, virologically suppressed patients. Expert Opin. Pharmacother..

[14] Margolis A.M., Heverling H., Pham P.A., Stolbach A. (2013). A review of the toxicity of HIV medications. J. Med. Toxicol..

[15] Ramsay R.R., Popovic-Nikolicb M.R., Nikolic K., Uliassi E., Bolognesi M.L. (2018). A perspective on multi-target drug discovery and design for complex diseases. Clin. Transl. Med..

[16] de Castro S., Camarasa M.-J. (2018). Polypharmacology in HIV inhibition: Can a drug with simultaneous action against two relevant targets be an alternative to combination therapy?. Eur. J. Med. Chem..

[17] Morphy R., Rankovic Z., Wermuth C.G. (2008). Chapter 27—Multi-target drugs: Strategies and challenges for medicinal chemists. The Practice of Medicinal Chemistry.

[18] Velázquez S., Alvarez R., San-Felix A., Jimeno M.L., De Clercq E., Balzarini J., Camarasa M.J. (1995). Synthesis and anti-HIV activity of [AZT]-[TSAO-T] and [AZT]-[HEPT] dimers as potential multifunctional inhibitors of HIV-1 reverse transcriptase. J. Med. Chem..

[19] Velázquez S., Tuñón V., Jimeno M.L., Chamorro C., De Clercq E., Balzarini J., Camarasa M.J. (1999). Potential multifunctional inhibitors of HIV-1 reverse transcriptase. Novel [AZT]-[TSAO-T] and [d4T]-[TSAO-T] heterodimers modified in the linker and in the dideoxynucleoside region. J. Med. Chem..

[20] Telesnitsky A., Goff S.P. (1997). Reverse Transcriptase and the Generation of Retroviral DNA.

[21] Nikolenko G.N., Palmer S., Maldarelli F., Mellors J.W., Coffin J.M., Pathak V.K. (2005). Mechanism for nucleoside analog-mediated abrogation of HIV-1 replication: Balance between RNase H activity and nucleotide excision. Proc. Natl. Acad. Sci. USA.

[22] Tisdale M., Schulze T., Larder B.A., Moelling K. (1991). Mutations within the RNase H domain of Human Immunodeficiency Virus Type 1 reverse transcriptase abolish virus infectivity. J. Gen. Virol..

[23] Schatz O., Cromme F.V., Grüninger-Leitch F., Le Grice S.F. (1989). Point mutations in conserved amino acid residues within the C-terminal domain of HIV-1 reverse transcriptase specifically repress RNase H function. FEBS Lett..

[24] Boyer P.L., Smith S.J., Zhao X.Z., Das K., Gruber K., Arnold E., Burke T.R., Hughes S.H. (2018). Developing and evaluating inhibitors against the RNase H active site of HIV-1 reverse transcriptase. J. Virol..

[25] Wang X., Gao P., Menendez-Arias L., Liu X., Zhan P. (2018). Update on recent developments in small molecular HIV-1 RNase H inhibitors (2013–2016): Opportunities and challenges. Curr. Med. Chem..

[26] Figiel M., Krepl M., Poznański J., Gołąb A., Sponer J., Nowotny M. (2017). Coordination between the polymerase and RNase H activity of HIV-1 reverse transcriptase. Nucleic Acids Res..

[27] Himmel D.M., Maegley K.A., Pauly T.A., Bauman J.D., Das K., Dharia C., Clark A.D., Ryan K., Hickey M.J., Love R.A. (2009). Structure of HIV-1 reverse transcriptase with the inhibitor β-thujaplicinol bound at the RNase H active site. Structure.

[28] Wendeler M., Lee H.-F., Bermingham A., Miller J.T., Chertov O., Bona M.K., Baichoo N.S., Ehteshami M., Beutler J., O’Keefe B.R. (2008). Vinylogous ureas as a novel class of inhibitors of reverse transcriptase-associated ribonuclease H activity. ACS Chem. Biol..

[29] Klumpp K., Mirzadegan T. (2006). Recent progress in the design of small molecule inhibitors of HIV RNase H. Curr. Pharm. Des..

[30] Himmel D.M., Sarafianos S.G., Dharmasena S., Hossain M.M., McCoy-Simandle K., Ilina T., Clark A.D., Knight J.L., Julias J.G., Clark P.K. (2006). HIV-1 reverse transcriptase structure with RNase H inhibitor dihydroxy benzoyl naphthyl hydrazone bound at a novel site. ACS Chem. Biol..

[31] Tramontano E., Corona A., Menéndez-Arias L. (2019). Ribonuclease H, an unexploited target for antiviral intervention against HIV and hepatitis B virus. Antivir. Res..

[32] Corona A., Ballana E., Distinto S., Rogolino D., Del Vecchio C., Carcelli M., Badia R., Riveira-Muñoz E., Esposito F., Parolin C. (2020). Targeting HIV-1 RNase H: *N’*-(2-hydroxy-benzylidene)-3,4,5-trihydroxybenzoylhydrazone as selective inhibitor active against NNRTIs-resistant variants. Viruses.

[33] Tramontano E., Esposito F., Badas R., Costi R., Di Santo R., Colla P. RDS1643, a novel diketo acid which selectively inhibits the HIV-1 multiplication in cell-based assays and the ribonuclease H activity in enzyme assays. Proceedings of the 17th International Conference on Antiviral Research.

[34] Distinto S., Esposito F., Kirchmair J., Cardia M.C., Gaspari M., Maccioni E., Alcaro S., Markt P., Wolber G., Zinzula L. (2012). Identification of HIV-1 reverse transcriptase dual inhibitors by a combined shape, 2D-fingerprint and pharmacophore-based virtual screening approach. Eur. J. Med. Chem..

[35] Schneider A., Corona A., Spöring I., Jordan M., Buchholz B., Maccioni E., Di Santo R., Bodem J., Tramontano E., Wöhrl B.M. (2016). Biochemical characterization of a multi-drug resistant HIV-1 subtype AG reverse transcriptase: Antagonism of AZT discrimination and excision pathways and sensitivity to RNase H inhibitors. Nucleic Acids Res..

[36] Sonar V.P., Corona A., Distinto S., Maccioni E., Meleddu R., Fois B., Floris C., Malpure N.V., Alcaro S., Tramontano E. (2017). Natural product-inspired esters and amides of ferulic and caffeic acid as dual inhibitors of HIV-1 reverse transcriptase. Eur. J. Med. Chem..

[37] Meleddu R., Distinto S., Corona A., Tramontano E., Bianco G., Melis C., Cottiglia F., Maccioni E. (2016). Isatin thiazoline hybrids as dual inhibitors of HIV-1 reverse transcriptase. J. Enzym. Inhib. Med. Chem..

[38] Corona A., Meleddu R., Esposito F., Distinto S., Bianco G., Masaoka T., Maccioni E., Menéndez-Arias L., Alcaro S., Le Grice S.F.J. (2016). Ribonuclease H/DNA polymerase HIV-1 reverse transcriptase dual inhibitor: Mechanistic studies on the allosteric mode of action of isatin-based compound RMNC6. PLoS ONE.

[39] Meleddu R., Distinto S., Corona A., Bianco G., Cannas V., Esposito F., Artese A., Alcaro S., Matyus P., Bogdan D. (2015). (3Z)-3-(2-[4-(aryl)-1,3-thiazol-2-yl]hydrazin-1-ylidene)-2,3-dihydro-1H-indol-2-one derivatives as dual inhibitors of HIV-1 reverse transcriptase. Eur. J. Med. Chem..

[40] Distinto S., Maccioni E., Meleddu R., Corona A., Alcaro S., Tramontano E. (2013). Molecular aspects of the RT/drug interactions. Perspective of dual inhibitors. Curr. Pharm. Des..

[41] Corona A., Meleddu R., Esposito F., Distinto S., Bianco G., Maccioni E., Le Grice S.S., Tramontano E. (2013). Site directed mutagenesis studies on HIV-1 reverse transcriptase (RT) shed light on the mechanism of action of a new ribonuclease H/ DNA polymerase RT dual inhibitor. Retrovirology.

[42] Esposito F., Mandrone M., del Vecchio C., Carli I., Distinto S., Corona A., Lianza M., Piano D., Tacchini M., Maccioni E. (2017). Multi-target activity of Hemidesmus indicus decoction against innovative HIV-1 drug targets and characterization of Lupeol mode of action. Pathog. Dis..

[43] Tramontano E., Esposito F., Badas R., Di Santo R., Costi R., La Colla P. (2005). 6-[1-(4-Fluorophenyl)methyl-1H-pyrrol-2-yl)]-2,4-dioxo-5-hexenoic acid ethyl ester a novel diketo acid derivative which selectively inhibits the HIV-1 viral replication in cell culture and the ribonuclease H activity in vitro. Antivir. Res..

[44] Sarafianos S.G., Das K., Hughes S.H., Arnold E. (2004). Taking aim at a moving target: Designing drugs to inhibit drug-resistant HIV-1 reverse transcriptases. Curr. Opin. Struct. Biol..

[45] Chung J.Y., Hah J.-M., Cho A.E. (2009). Correlation between performance of QM/MM docking and simple classification of binding sites. J. Chem. Inf. Model..

[46] Ellingson S.R., Miao Y., Baudry J., Smith J.C. (2014). Multi-conformer ensemble docking to difficult protein targets. J. Phys. Chem. B.

[47] Ren J., Esnouf R., Garman E., Somers D., Ross C., Kirby I., Keeling J., Darby G., Jones Y., Stuart D. (1995). High-resolution structures of HIV-1 RT from four RT–inhibitor complexes. Nat. Struct. Mol. Biol..

[48] Das K., Bauman J.D., Clark A.D., Frenkel Y.V., Lewi P.J., Shatkin A.J., Hughes S.H., Arnold E. (2008). High-resolution structures of HIV-1 reverse transcriptase/TMC278 complexes: Strategic flexibility explains potency against resistance mutations. Proc. Natl. Acad. Sci. USA.

[49] Ren J., Nichols C., Bird L.E., Fujiwara T., Sugimoto H., Stuart D., Stammers D.K. (2000). Binding of the second-generation non-nucleoside inhibitor S-1153 to HIV-1 reverse transcriptase involves extensive main chain hydrogen bonding. J. Biol. Chem..

[50] Das K., Bauman J.D., Rim A.S., Dharia C., Clark A.D., Camarasa M.J., Balzarini J., Arnold E. (2011). Crystal structure of tert-butyldimethylsilyl-spiroaminooxathioledioxide-thymine (TSAO-T) in complex with HIV-1 reverse transcriptase (RT) redefines the elastic limits of the non-nucleoside inhibitor-binding pocket. J. Med. Chem..

[51] Pata J., Stirtan W.G., Goldstein S.W., Steitz T.A. (2004). Structure of HIV-1 reverse transcriptase bound to an inhibitor active against mutant reverse transcriptases resistant to other nonnucleoside inhibitors. Proc. Natl. Acad. Sci. USA.

[52] Su H.-P., Yan Y., Prasad G.S., Smith R.F., Daniels C.L., Abeywickrema P.D., Reid J.C., Loughran H.M., Kornienko M., Sharma S. (2010). Structural basis for the inhibition of RNase H activity of HIV-1 reverse transcriptase by RNase H active site-directed inhibitors. J. Virol..

[53] Corona A., Di Leva F.S., Thierry S., Pescatori L., Crucitti G.C., Subra F., Delelis O., Esposito F., Rigogliuso G., Costi R. (2014). Identification of highly conserved residues involved in inhibition of HIV-1 RNase H function by diketo acid derivatives. Antimicrob. Agents Chemother..

[54] Suchaud V., Bailly F., Lion C., Tramontano E., Esposito F., Corona A., Christ F., Debyser Z., Cotelle P. (2012). Development of a series of 3-hydroxyquinolin-2(1H)-ones as selective inhibitors of HIV-1 reverse transcriptase associated RNase H activity. Bioorganic Med. Chem. Lett..

[55] Costa G., Rocca R., Corona A., Grandi N., Moraca F., Romeo I., Talarico C., Gagliardi M.G., Ambrosio F.A., Ortuso F. (2019). Novel natural non-nucleoside inhibitors of HIV-1 reverse transcriptase identified by shape- and structure-based virtual screening techniques. Eur. J. Med. Chem..

[56] Mohamadi F., Richards N.G.J., Guida W.C., Liskamp R., Lipton M., Caufield C., Chang G., Hendrickson T., Still W.C. (1990). Macromodel—An integrated software system for modeling organic and bioorganic molecules using molecular mechanics. J. Comput. Chem..

[57] Halgren T.A. (1996). Merck molecular force field. II. MMFF94 van der Waals and electrostatic parameters for intermolecular interactions. J. Comput. Chem..

[58] Still W.C., Tempczyk A., Hawley R.C., Hendrickson T. (1990). Semianalytical treatment of solvation for molecular mechanics and dynamics. J. Am. Chem. Soc..

[59] (2021). QM-Polarized Protocol.

[60] Wolber G., Dornhofer A.A., Langer T. (2006). Efficient overlay of small organic molecules using 3D pharmacophores. J. Comput. Mol. Des..

[61] Wolber G., Langer T. (2004). LigandScout: 3-D pharmacophores derived from protein-bound ligands and their use as virtual screening filters. J. Chem. Inf. Model..

